# Alcohol consumption, drinking patterns, and ischemic heart disease: a narrative review of meta-analyses and a systematic review and meta-analysis of the impact of heavy drinking occasions on risk for moderate drinkers

**DOI:** 10.1186/s12916-014-0182-6

**Published:** 2014-10-21

**Authors:** Michael Roerecke, Jürgen Rehm

**Affiliations:** Social and Epidemiological Research Department, Centre for Addiction and Mental Health (CAMH), 33 Russell Street, Toronto, Ontario M5S 2S1 Canada; Dalla Lana School of Public Health (DLSPH), University of Toronto, Toronto, Canada; Institute of Medical Science, University of Toronto, Toronto, Canada; Institute for Clinical Psychology and Psychotherapy, TU Dresden, Dresden, Germany; Department of Psychiatry, University of Toronto, Toronto, Canada

**Keywords:** Alcohol, Binge drinking, Heavy drinking, Ischemic heart disease, Meta-analysis, Systematic review

## Abstract

**Background:**

Alcohol consumption is a major global risk factor for mortality and morbidity. Much discussion has revolved around the diverse findings on the complex relationship between alcohol consumption and the leading cause of death and disability, ischemic heart disease (IHD).

**Methods:**

We conducted a systematic search of the literature up to August 2014 using Preferred Reporting Items for Systematic Reviews and Meta-Analyses guidelines to identify meta-analyses and observational studies examining the relationship between alcohol drinking, drinking patterns, and IHD risk, in comparison to lifetime abstainers. In a narrative review we have summarized the many meta-analyses published in the last 10 years, discussing the role of confounding and experimental evidence. We also conducted meta-analyses examining episodic heavy drinking among on average moderate drinkers.

**Results:**

The narrative review showed that the use of current abstainers as the reference group leads to systematic bias. With regard to average alcohol consumption in relation to lifetime abstainers, the relationship is clearly J-shaped, supported by short-term experimental evidence and similar associations within strata of potential confounders, except among smokers. Women experience slightly stronger beneficial associations and also a quicker upturn to a detrimental effect at lower levels of average alcohol consumption compared to men. There was no evidence that chronic or episodic heavy drinking confers a beneficial effect on IHD risk. People with alcohol use disorder have an elevated risk of IHD (1.5- to 2-fold). Results from our quantitative meta-analysis showed that drinkers with average intake of <30 g/day and no episodic heavy drinking had the lowest IHD risk (relative risk = 0.64, 95% confidence interval 0.53 to 0.71). Drinkers with episodic heavy drinking occasions had a risk similar to lifetime abstainers (relative risk = 1.12, 95% confidence interval 0.91 to 1.37).

**Conclusions:**

Epidemiological evidence for a beneficial effect of low alcohol consumption without heavy drinking episodes is strong, corroborated by experimental evidence. However, episodic and chronic heavy drinking do not provide any beneficial effect on IHD. Thus, average alcohol consumption is not sufficient to describe the risk relation between alcohol consumption and IHD. Alcohol policy should try to reduce heavy drinking patterns.

**Electronic supplementary material:**

The online version of this article (doi:10.1186/s12916-014-0182-6) contains supplementary material, which is available to authorized users.

## Background

Ischemic heart disease (IHD) is the leading cause of death and disease burden in the US [1], Europe [2], and globally [3,4], and alcohol consumption is one of the leading risk factors for mortality and morbidity [5,6]. There are well-established risks from neuro-toxic, hepato-toxic, and carcinogenic effects caused by alcohol consumption (for example, the risk for cancers of the upper aerodigestive tract [7–9], injuries [6,10], and liver cirrhosis [6,10,11]). However, there has been much debate about a beneficial effect of alcohol consumption on IHD [12–14]. High prevalence of both exposure and disease make this question a frequent topic among general practitioners, researchers, media, and the public. Aside from numerous individual studies, several meta-analyses published in the last decade have summarized the association between alcohol consumption and IHD risk.

Most meta-analyses of epidemiological data have shown a mix between a beneficial and detrimental association from alcohol consumption on IHD that depends on the level of average consumption. This relationship is most often described as curvilinear, or ‘J-shaped’ [15,16], but also sometimes as a flattened-out inverse association [15,17,18]. The specific shape of the risk curve seems to depend at least on sex and IHD outcome (mortality versus morbidity). Findings of a beneficial effect are supported by a substantial number of short-term experimental studies on the effect of alcohol consumption on several surrogate biomarkers for IHD in a dose-dependent relationship [19,20], including improved lipid profiles, inhibition of platelet activation, reduction of fibrinogen levels, and anti-inflammatory effects. In particular, high density lipoprotein (HDL) cholesterol levels have a clear dose-response relationship with alcohol consumption, with the highest levels observed in people with the highest alcohol consumption [21,22]. Many criticisms have arisen over the last three decades questioning the relationship found in epidemiological studies because of limited quality of alcohol assessment, the influence of drinking pattern, adjustment for confounding, or the inability for observational studies to determine causality [13,23]. Although criteria for a causal relationship [24] seem to be fulfilled (see also [15,18]), a direct link for alcohol consumption on IHD risk from long-term randomized trials is currently, and for the foreseeable future, missing. Thus, epidemiological studies, as is the case for many other IHD risk factors, play an important role in assessing the role of alcohol consumption on disease risk. The objective of this review is to examine the evidence available to define the relationship between alcohol consumption and IHD based mainly on systematic reviews and meta-analyses, with a focus on the reference group (that is, the use of lifetime abstainers and not current abstainers as the reference group because of the ‘sick-quitter’ effect [25]); the influence of drinking pattern (in particular episodic heavy drinking among on average moderate drinkers [26]); and the influence of several other important risk factors for IHD, such as age, smoking status, physical activity, and body mass index (BMI), all of which might confound risk estimates for alcohol.

## Methods

### Searches

Using Preferred Reporting Items for Systematic Reviews and Meta-Analyses (PRISMA) guidelines [27], we conducted two systematic searches in electronic databases from 1980 up to second week of August. First, we searched electronic databases for meta-analyses on alcohol consumption and IHD risk. Second, we searched for original articles, excluding letters, editorials, conference abstracts, reviews, and comments, for variations of search terms for the exposure (alcohol consumption), outcome (IHD), and study design based on previous meta-analyses [15,26]. For details, please see Additional file 1: Text S1, Figures S1 and S2. Additionally, we hand-searched references of identified papers and relevant reviews and meta-analyses. Using articles reviewed in these two searches, we examined the role of the reference group, average alcohol consumption, drinking patterns, confounders, and experimental evidence on the alcohol-IHD relationship in a narrative review, and conducted a quantitative analysis on drinking patterns among on average moderate alcohol drinkers in relation to lifetime abstention.

### Meta-analysis

#### Inclusion and exclusion criteria

Inclusion criteria for a quantitative analysis of drinking patterns in relation to lifetime abstainers were as follows: adult (≥18 years) population samples; IHD analyzed as a separate outcome (International Classification of Diseases (ICD)-9: 410-414, ICD-10: I20-25); case-control, prospective, or historical cohort study design; alcohol exposure measurement covering a reference period of more than 2 weeks for average alcohol consumption at baseline; a drinking group that either specifically excluded or included episodic heavy drinking among current drinkers with an average alcohol consumption <30 g of pure alcohol per day; a measure of risk in comparison to lifetime abstainers and its corresponding measure of variability was reported (or sufficient data to calculate these); and English, German, or Spanish language. We excluded self-reported IHD outcomes and samples from people with IHD-related conditions.

#### Data abstraction

For the meta-analyses on drinking patterns in reference to lifetime abstention, we extracted from all relevant articles authors’ names, year of publication, country, calendar year(s) of baseline examination, follow-up period, setting, assessment of IHD and alcohol consumption, mean and range of age at baseline, sex, number of observed IHD cases or deaths among participants by drinking group, number of total participants by drinking group, adjustment for potential confounders, and relative risk (RR) and its standard error. We used the most adjusted RR reported, and gave priority to estimates comparing drinking to lifetime abstainers. Information found in related papers from the same cohort was used where possible. The first author performed the literature search and abstracted the data. Full-text articles with potential eligibility were discussed by both authors until consensus was reached. Primary authors were not contacted where there was not enough information presented in the article.

#### Statistical analysis

Hazard ratios, odds ratios, and RRs were treated as equivalent measures of risk. If necessary, RRs within studies were re-calculated based on the method described by Hamling *et al*. [28] and pooled across studies using inverse-variance weighted DerSimonian-Laird random-effect models to allow for between-study heterogeneity [29]. We quantified between-study heterogeneity using Cochran’s Q [30] and the I^2^ statistic [31]. I^2^ can be interpreted as the proportion of the total variation other than chance that is due to heterogeneity between studies. We tested for potential publication bias using Egger’s test [32]. Sensitivity analyses for the influence of single studies on the pooled RRs were conducted omitting one study at a time and re-estimating the pooled RR. All meta-analytical procedures were conducted on the natural log scale in Stata statistical software, version 12.1 (Stata Corp, College Station, TX, USA), and *P* <0.05 (two-sided) was considered statistically significant.

## Results and discussion

In the following paragraphs, we describe the results of previous systematic reviews and meta-analyses, and individual studies on alcohol consumption and IHD risk in a narrative review. Furthermore, we meta-analyze the role of heavy drinking patterns in reference to lifetime abstainers using high-quality observational studies.

### Lifetime abstainers and former drinkers

The majority of studies on alcohol consumption and IHD used current abstainers (that is, no current alcohol intake and no assessment of past alcohol intake) as the reference group and thus did not distinguish between lifetime abstainers and former drinkers. For almost 30 years this has been the most important question about the validity of epidemiological findings on the alcohol-IHD relationship. Shaper and colleagues put forward the concept of a ‘sick-quitter’ to describe the elevated risk of many current abstainers and former drinkers for health outcomes [25]. A recent systematic investigation using evidence from 54 epidemiological studies showed that former drinkers were at higher risk for IHD mortality [33]. The pooled IHD mortality risk among former drinkers was 1.54 (95% confidence interval (CI) 1.17 to 2.03) in women, and 1.25 (1.15 to 1.36) in men in comparison to lifetime abstainers. The definition of lifetime abstainers (for example, whether less than 12 drinks over the lifetime or very infrequent drinking over the lifetime with no more than 12 drinks in a single year) did not influence the conclusions about this effect. However, it should be noted that a distinction between former drinkers and lifetime abstainers might not be enough to accurately describe IHD risk among current non-drinkers. Rogers *et al*. [34] found that non-drinkers have different reasons for not drinking and that there is evidence for heterogeneity among non-drinkers that might not be captured completely by dividing non-drinkers into lifetime abstainers and former drinkers.

### Average alcohol consumption

Another recent meta-analysis [15] presented the risk of current drinkers by level of average alcohol intake in comparison to lifetime abstainers where those estimates were available and simultaneously adjusting studies using current abstainers (that is, compensating for the elevated risk in former drinkers) based on the above-mentioned meta-analysis. The results clearly showed evidence for a beneficial effect when all available studies were included regardless of sex and IHD outcome (incidence, mortality, or morbidity). In particular, all pooled IHD risk estimates were statistically significant for average alcohol consumption of one to two drinks per day (point estimates were between 0.69 and 0.81 in comparison to lifetime abstainers). The results also showed that the particular J-shape of the association differed by sex and IHD outcome in stratified analyses. Sex seems to be important in that women experience slightly stronger beneficial associations and also a quicker upturn to a detrimental effect at lower levels of average alcohol consumption compared to men [15], which might be related to sex-specific biological factors, such as of body fat distribution, body size, and alcohol solubility [35–37].

Although some meta-analyses [16,18,38] have reported a protective association even for chronic heavy alcohol consumers in population studies (total alcohol intake on average ≥60 g pure alcohol/day), these results need to be interpreted with caution because the reference group is of crucial importance, as shown above. The association seems beneficial among chronic heavy drinkers only when the reference group comprises current abstainers (that is, lifetime abstainers and former drinkers). For example, Ronksley *et al*. [18] reported a pooled RR of 0.76 (95% CI 0.52 to 1.09) for IHD incidence and 0.75 (95% CI 0.63 to 0.89) for IHD mortality among chronic drinkers consuming ≥60 g/day in comparison to current non-drinkers.

**Figure 1:**
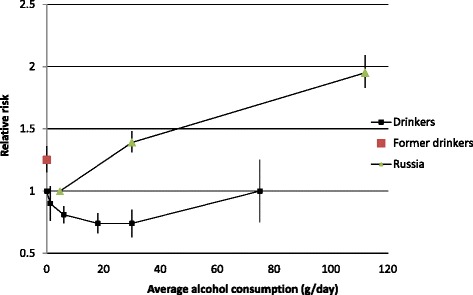
**The association of ischemic heart disease mortality with average alcohol consumption in comparison to lifetime abstention in men.** Data points taken from published meta-analyses [15,33,39]. All point estimates and confidence intervals were obtained from categorical meta-analyses stratified by alcohol exposure. The Russian estimates were pooled from Zaridze *et al*. [40,41].

Most recently, a systematic review and meta-analysis [39] demonstrated that chronic heavy drinking does not show any beneficial association with IHD risk when lifetime abstainers are the reference group. IHD mortality risk among male chronic heavy drinkers (≥60 g/day) was similar to lifetime abstainers with no indication for a protective association (RR = 1.00, 95% CI 0.74 to 1.36). Similarly, IHD incidence (that is, using both mortality and morbidity outcomes) showed no indication of a protective effect (RR = 1.04, 95% CI 0.83 to 1.31) [39]. Such chronic heavy drinking is rarely observed in women in population studies and there are not enough studies to systematically investigate chronic heavy drinking compared to lifetime abstention in women. The above-mentioned IHD mortality risks of average alcohol consumption among men in comparison to lifetime abstainers using data from several previous meta-analyses are displayed in Figure 1.

Whereas the aforementioned investigations were conducted using data from population studies, evidence from clinical samples involving patients with alcohol use disorder (AUD) in alcohol treatment showed a detrimental association with IHD mortality in both men and women (RR = 1.62, 95% CI 1.34 to 1.95 in men and RR = 2.09, 95% CI 1.28 to 3.41 in women compared to the general population, see also [42]) in a recent meta-analysis [39]. Patients with AUD are typically missed or underrepresented in population studies [43].

Among those reporting the strongest elevated risk for IHD are studies from Russia [40,41]. These studies consistently report substantially elevated RRs in heavy drinkers; however, alcohol consumption seems so prevalent in Russia that there have not been enough lifetime abstainers to define the risk relationship in comparison to zero alcohol intake over the life course. Nevertheless, the risk among heavy alcohol drinkers in comparison to low-level drinkers [40,41] was substantial (Figure 1).

Although no reliable comparisons exist because lifetime abstention is rare in Russia, one can speculate whether the estimates for heavy drinking are over- or underestimates compared to lifetime abstainers. Assuming the reference group (0.2 half-liter bottles of vodka per week or 4.6 g/day on average) has a similar risk compared to low-level drinkers elsewhere (RR = 0.81), the adjusted risk would be 1.58 (95% CI 1.48 to 1.69), only slightly less than assuming the risk among on average low-level drinkers in Russia is indeed equal to that of lifetime abstainers elsewhere (RR = 1.00, Figure 1). However, given the heavy episodic drinking pattern common in Russia, one would not necessarily expect to find any beneficial effect from any alcohol consumption on IHD risk on a population level as we argue below. Similarly, if one assumes a beneficial effect from average moderate alcohol consumption for 25% of the population, the risk in male patients with AUD would be slightly less (RR = 1.36, 95% CI 1.13 to 1.64). In summary, the relationship between average alcohol consumption and IHD risk is clearly J-shaped with an increased IHD risk at high levels of alcohol consumption when compared to lifetime abstainers or low-level drinkers.

### Drinking pattern

Alcohol can be consumed in many different ways, leading to the concern that an episodic heavy drinking pattern may confound or modify the relationship seen for average volume of alcohol intake and IHD risk [44,45]. McElduff and Dobson were the first to present a stratified risk matrix by amount of alcohol consumption on drinking days and frequency of such drinking days, in the Australian part of the MONICA project looking at myocardial infarction risk [46]. Since then, several other studies have examined the alcohol-IHD relationship with similar detail, making it possible to investigate the influence of drinking patterns more systematically. Excluding the potential problem of lifetime abstainers and former drinkers, a recent meta-analysis examined drinking patterns among current drinkers who were not chronic heavy drinkers (that is, excluding those with average total alcohol intake of ≥60 g/day) [26]. This meta-analysis found a significant difference when comparing episodic heavy drinkers with moderate regular drinkers, with a pooled RR = 1.45 (95% CI 1.24 to 1.70). Other studies published since then have shown similar findings [33,47].

#### Meta-analysis on drinking pattern among moderate alcohol drinkers

**Figure 2:**
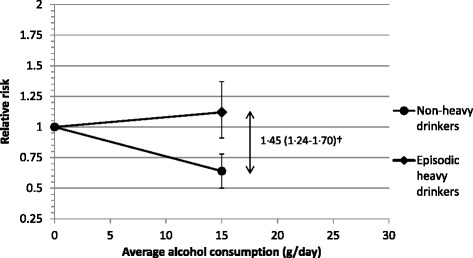
**Ischemic heart disease incidence by drinking pattern among drinkers with average consumption of <30 g/day in comparison to lifetime abstention.** Please see Additional file 1: Table S1, Figures S3 and S4 for details. ^†^Taken from Roerecke & Rehm [26].

In a quantitative meta-analysis paying special attention to the effects of alcohol consumption patterns, we systematically examined IHD risk among two distinct drinking groups with the same average alcohol intake (Figure 2). We identified seven studies providing data on episodic heavy drinking at low to moderate average alcohol consumption (<30 g/day) in comparison to lifetime abstainers (Additional file 1: Table S1, Figures S3 and S4). Compared with lifetime abstainers (that is, not including former drinkers), the pooled RR for IHD incidence was 0.64 (95% CI 0.53 to 0.71) for moderate drinkers without heavy drinking occasions, and 1.12 (95% CI 0.91 to 1.37) for drinkers with the same average amount who engaged in heavy episodic drinking (Figure 2, Additional file 1: Table S1, Figures S3 and S4). There was no evidence for publication bias (*P* = 0.35 and 0.58 for moderate non-heavy drinkers and episodic heavy drinkers, respectively). None of the primary studies in the two meta-analyses had a large influence on the pooled RR estimates. Furthermore, there was very little heterogeneity (10% and 0%, respectively). All studies were adjusted for age and smoking status, five for education and other indicators for socio-economic status, and four each for BMI and marital status.

The corresponding RR between these two drinking groups was 1.75 (95% CI 1.36 to 2.25), higher than the estimate from the previous meta-analysis [26]. In other words, the impact of episodic heavy drinking seems to be greatest at low levels of average alcohol consumption in studies that have separated lifetime abstainers from former drinkers and were well-adjusted for the most relevant potential confounders (Additional file 1: Table S1). Furthermore, the risk estimate for non-heavy low-level drinking was lower (that is, stronger in magnitude for a beneficial effect) than previous investigations of average alcohol consumption without taking into account episodic heavy drinking occasions [15,18].

#### Studies from Russia

The importance of drinking patterns becomes particularly important when looking at Russian studies [40,48–50]. A relatively frequent consumption pattern in Russia is episodic heavy to very heavy consumption with sometimes prolonged binges (‘zapoi’, an episode of continuous drunkenness lasting two or more days in combination with withdrawal from normal social life [51]). This drinking pattern is so extreme that it is heavy with regard to both average and episodic consumption. For example Malyutina *et al.* [48] in the Russian component of the MONICA project reported that only 7% of their sample drank 40 g pure alcohol or less per typical occasion. Moreover, 12% of this Russian sample were current abstainers, 55% reported drinking 80 g or more per typical occasion, and only 8% had a drinking frequency of more than two days per week. In comparison, the National Health Interview Survey cohort from the US had 16 % lifetime abstainers, 15% former drinkers, 42% infrequent or moderate drinkers, and only 27% of the participants drank three or more drinks (≥36 g pure alcohol) per drinking day [52].

There is substantial epidemiological evidence showing no protective effect on IHD risk from episodic heavy drinking, whereas the evidence for a beneficial effect of alcohol is substantial and strongest among non-heavy low-level drinkers. In summary, drinking patterns have modifying effects on the relationship between average alcohol consumption and IHD risk.

### Confounding from other risk factors for ischemic heart disease

Residual confounding is an issue for all risk factors for IHD in observational studies. Many risk factors for IHD have been identified [1]. Inclusion of potential confounders had little influence on the pooled risk estimates from meta-analyses examining drinking versus non-drinking status [18]; this finding was similar within categories of average alcohol consumption in a pooled individual-data analysis of eight cohort studies (confounders included age; year of baseline; smoking; BMI; education; physical activity; energy intake; intake of polyunsaturated fat, monounsaturated fat, saturated fat, fiber, and cholesterol; and study design) [53]. Aside from adjustment for confounding, many studies have reported stratified analyses by important risk factors for IHD, which we detail below.

#### Age

Hvidtfeldt *et al*., in a pooled individual-level analysis, showed an inverse relationship for each sex and each of three age groups based on eight cohort studies with 250,000 participants [53]. An inverse relationship based on 64,000 participants stratified into below 60 years of age and 60 years or above has been shown in Chinese men [54]. An analysis of the male British Doctors cohort found an inverse relationship both among participants younger than 75 years and those 75 years and above [55]. In a case-control study from Japan, Miyake found an inverse relationship both among participants younger than 65 years and those 65 years and above [56]. A case-control study from Portugal showed a U-shape in those under 45 years of age and an elevated risk only in study participants drinking more than 60 g of alcohol per day among participants 45 years or older [57]. The Honolulu Heart Program cohort showed an inverse relationship both among participants aged between 51 and 65 years and those 65 to 75 years old [58].

#### Smoking

Although numerous modifiable risk factors for IHD have been identified, their influence on the alcohol-IHD relationship seems to be small, except for smoking. Smoking is, aside from age, the most important risk factor for IHD, and several studies have provided evidence on its influence on the alcohol-IHD relationship. It should be noted that alcohol is one of the most investigated risk factors for IHD [59]. Inoue *et al.*, in a pooled individual analysis by smoking status, showed a J-shape in never smokers and a U-shape in current smokers, with the highest category of average alcohol consumption being 92 g/day or more in 300,000 Japanese participants [60]. A Chinese cohort study showed a similar inverse relationship in both current smokers and current non-smokers [54]. An inverse relationship among never smokers, a U-shape in former smokers, and an exponential relationship in current smokers was reported in a male Scottish sample of factory workers between 35 and 64 years old and with 30 years of follow-up [61]. Ebbert *et al*. showed an inverse relationship among never smokers and former smokers, and no relationship among current smokers in a low consumption cohort - the Iowa Women’s Health study [62]. An analysis of the Framingham study with 24 years of follow-up showed an inverse relationship among non-smokers, no relationship among light smokers (≤1 pack/day), and an inverse relationship among heavy smokers (>1 pack/day) in men. In women, a U-shape was found among non-smokers and smokers [63]. An analysis of the British Regional Heart Study showed an inverse relationship among former smokers, no relationship among current smokers, and an unclear relationship among never smokers. However, there were too few IHD deaths among never drinkers to reach a firm conclusion [64]. In an investigation of the National Health and Nutrition Examination Survey (NHANES I) in women 45 to 74 years old, an inverse relationship in both smokers and non-smokers was reported [65].

In sum, regarding average alcohol consumption, in all but one population study an inverse or J-shaped curve was observed in never or non-smokers. Evidence in smokers is mixed. Some studies reported an inverse relationship, some a threshold relationship, and some no clear relationship. Regarding clinical samples of patients in AUD treatment, there is the possibility that the detrimental association from alcohol consumption is overestimated because of uncontrolled confounding from smoking in these samples. However, the prospective Russian study by Zaridze *et al.* [41] clearly showed a substantially increasing risk with increasing alcohol consumption among male smokers.

#### Other confounding factors

Some evidence stratified by physical activity and BMI for the alcohol-IHD relationship exists. Pedersen *et al.* investigated fatal IHD in the Copenhagen City Heart Study [66]. They found an inverse relationship for both physical activity level and average alcohol consumption in a low consumption cohort. The risk for non-drinkers and drinkers having less than one drink per week was consistently higher compared to drinkers having one to 14 drinks per week and 15 or more drinks. They concluded that both physical activity and alcohol consumption were factors for lower IHD risk. Bazzano *et al*. found an inverse relationship for participants with a BMI ≥25 and <25, with stronger evidence among those with a BMI <25 [54].

In sum, the epidemiological evidence shows that only in smokers there is some evidence that there is no beneficial association of alcohol consumption, and possibly a threshold effect, pointing to possible effect modification with alcohol consumption. Evidence for a beneficial association was consistent across age groups and in non-smokers. Available evidence for the influence of physical activity and BMI is sparse, although this evidence points to a beneficial association, as well. Furthermore, a beneficial association has been observed in patients who are hypertensive, diabetic, have cardiovascular diseases, and who are survivors of myocardial infarction [67–73]. An inverse relationship has been observed in healthy individuals in a US cohort [74], and no association in a UK cohort [75].

#### Experimental evidence

Long-term randomized studies on alcohol exposure and IHD mortality or morbidity in the general population are unavailable. Regular alcohol intake has been found to have beneficial, dose-dependent effects on surrogate biomarkers for IHD risk in short-term experimental studies, mainly by increasing HDL cholesterol levels, inhibiting platelet activation, reducing fibrinogen levels, and producing anti-inflammatory effects [19,76]. The increase in HDL cholesterol was also evident in experimental studies with regular heavy drinking (≥60 g/day every day) [77–82], and the highest levels of HDL cholesterol are found in people with AUD [21]. Despite elevated levels of HDL cholesterol even in regular heavy alcohol consumers [83], an increase in low-density lipoprotein (LDL) and other detrimental effects of episodic and chronic heavy alcohol consumption on heart disease risk seem to negate those beneficial effects, resulting in an overall neutral or detrimental association. The detrimental effect on blood pressure and arrhythmias [44,84–90] and atrial fibrillation [44,89,91–93], in particular from episodic and chronic heavy drinking, might play a role here, in combination with anti-atherosclerotic and anti-thrombotic processes. Although systematic experimental evidence for the effect of episodic heavy drinking is limited, the biochemical effects might involve HDL and LDL cholesterol levels, arrhythmias, and thrombosis [45]. It seems that episodic heavy drinking increases LDL cholesterol levels without a favorable effect on HDL [45], and possibly a transient detrimental effect on thrombosis, hypertension, and arrhythmias [44,45]. Prolonged chronic heavy drinking can result in the most extreme form of cardiac tissue damage, cardiomyopathy [94].

Recently, using Mendelian randomization, Holmes *et al.* [95] examined the impact of alcohol dehydrogenase 1B alleles on IHD risk, and concluded that for every level of alcohol consumption, an increase in average consumption was associated with an increase in IHD risk, that is, no protective effect. The rs1229984 A-allele is associated with lower alcohol consumption due to negatively experienced effects (including a flushing response) caused by the fast metabolization of alcohol into acetaldehyde [96]. This study design can be seen as quasi-randomized, assuming the rs1229984 A-allele is randomly distributed in the population, thus non-carriers having the same IHD risk aside from the effect of the rs1229984 A-allele. An additional assumption has to be for conclusions regarding the absence of a protective effect that the effect of the polymorphism is entirely mediated via average alcohol consumption. However, Holmes *et al.* showed that, aside from the association with lower average alcohol consumption, the rs1229984 A-allele was also related to less binge drinking. As we have shown above, both average alcohol consumption and drinking patterns interact in a complex way with regard to IHD risk, and the alcohol-heart relationship cannot be accurately described using only one of the dimensions of alcohol intake. Taken together, it is difficult to compare the results from Holmes *et al*. to the epidemiological literature we have described here. Nevertheless, Mendelian randomization studies may become a very useful tool in widening the evidence base for a causal relation between alcohol consumption and IHD risk, together with both observational and experimental studies on the specific effects of drinking patterns on the heart (please see also [97]).

Thus, there is substantial experimental evidence for a beneficial effect of low to moderate regular alcohol consumption on IHD, which disappears for episodic heavy drinking. Novel study designs may help in improving knowledge of the complex relationship between alcohol and IHD risk.

## Conclusions

Alcohol’s effect on the human body and mind is quite strong, even at low doses [98]. Its neuro-toxic, hepato-toxic, and carcinogenic properties make it a potent risk factor for disease burden. However, its effect on IHD risk also makes it an intriguing and sometimes controversial topic in disease epidemiology and public policy. The quality of epidemiological studies has substantially improved over the last three decades. Using current abstainers as the reference group leads to systematic bias and erroneous conclusions. Using high-quality epidemiological evidence, a clear picture supported by short-term experimental evidence emerges. When examining average alcohol consumption in comparison to lifetime abstainers, the relationship with IHD risk follows a J-curve. The curve turns into a detrimental association for much lower average alcohol levels in women compared with men.

However, average alcohol consumption alone is not sufficient to describe the alcohol-IHD relationship. Drinking patterns play an important role and both episodic and chronic heavy drinking negate any beneficial association with IHD risk, or elevate the risk substantially. Nevertheless, for drinkers having one to two drinks per drinking day without episodic heavy drinking, there is substantial and consistent evidence from epidemiological and short-term experimental studies for a beneficial association with IHD risk when compared to lifetime abstainers. The alcohol-IHD relationship fulfills all criteria for a causal association proposed by Hill [24]. Whether one is able to detect an inverse, U-shaped, or J-shaped relationship depends on the distribution of drinking pattern in a given population. Prevalence of heavy drinking patterns has been on the rise in many countries, such as Canada, the US, the UK, and many Eastern European and Asian countries [99–102]. In the US, episodic heavy drinking is more common than chronic heavy drinking [102].

Aside from any effect on IHD, caution must be used when judging the overall risk-benefit relationship of any form of alcohol consumption on an individual level because of well-known detrimental effects on other disease outcomes, such as injuries and cancer [6,7,103]. Recommendations for clinical practitioners (aside from clear contra-indications because of other illnesses or medication intake) remain challenging because of the apparent simultaneous beneficial and detrimental effects from on average low alcohol consumption, and the fact that evidence from randomized controlled trials on long-term effects of alcohol consumption is and will be unavailable. Furthermore, there is no control mechanism for alcohol purchase as there is for prescription drugs because alcohol is freely available for self- and over-medication. Therefore, uptake of alcohol consumption should not be considered as a treatment option in prevention of IHD. In terms of public alcohol policy, the picture is clear: alcohol consumption should be as low as possible, no amount of consumption is safe, and any type of episodic and chronic heavy drinking should be strongly discouraged [104,105].

## Additional file

Additional file 1: Text S1. Systematic review protocols for narrative and quantitative reviews. **Figure S1.** Search results for meta-analyses on alcohol consumption and IHD risk. **Figure S2.** Search results for population studies on non-heavy and heavy alcohol consumption and IHD risk. **Table S1.** Characteristics of seven studies on IHD risk in people with 1 to 30 g/day average alcohol consumption in comparison to lifetime abstainers. **Figure S3.** IHD risk among non-heavy drinkers with total average intake of 1 to 30 g/day compared with lifetime abstainers. **Figure S4.** IHD risk among episodic heavy drinkers with total average intake of 1 to 30 g/day compared with lifetime abstainers.

## Abbreviations

AUD: alcohol use disorder
BMI: body mass index
CI: confidence interval
HDL: high-density lipoprotein
ICD: International Classification of Diseases
IHD: ischemic heart disease
LDL: low-density lipoprotein
RR: relative risk
