# Supplementary material for: Alcohol consumption, drinking patterns, and ischemic heart disease: a narrative review of meta-analyses and a systematic review and meta-analysis of the impact of heavy drinking occasions on risk for moderate drinkers
Source: BMC Med. 2014 Oct 21;12:182. doi: 10.1186/s12916-014-0182-6 (PMC4203905; doi:10.1186/s12916-014-0182-6)
Supplement: Additional file 1: Text S1. — Systematic review protocols for narrative and quantitative reviews. Figure S1. Search results for meta-analyses on alcohol consumption and IHD risk. Figure S2. Search results for population studies on non-heavy and heavy alcohol consumption and IHD risk. Table S1. Characteristics of seven studies on IHD risk in people with 1 to 30 g/day average alcohol consumption in comparison to lifetime abstainers. Figure S3. IHD risk among non-heavy drinkers with total average intake of 1 to 30 g/day compared with lifetime abstainers. Figure S4. IHD risk among episodic heavy drinkers with total average intake of 1 to 30 g/day compared with lifetime abstainers. [file 12916_2014_182_MOESM1_ESM.pdf]

Additional files

Supplement to: Roerecke M & Rehm J. Alcohol consumption, drinking patterns, and ischaemic heart disease: a narrative review of meta-analyses and a systematic review and meta-analysis of the impact of heavy drinking occasions on risk for moderate drinkers.

Content list

Text S1. Systematic review protocols for narrative and quantitative reviews

Figure S1. Search results for meta-analyses on alcohol consumption and IHD risk

Figure S2. Search results for population studies on non-heavy and heavy alcohol consumption and IHD risk

Table S1. Characteristics of 7 studies on IHD risk in drinkers of 1-30 g/day average alcohol consumption in comparison to lifetime abstainers

Figure S3. IHD risk among non-heavy drinkers with total average intake of 1-30 g/day compared to lifetime abstainers

Figure S4. IHD risk among episodic heavy drinkers with total average intake of 1-30 g/day compared to lifetime abstainers

Text S1. Systematic review protocols for narrative and quantitative reviews

Title: Systematic review and meta-analysis of alcohol drinking and ischaemic heart disease (IHD)

Protocol information

Dates

Systematic review was conducted in March 2014 (search 1 was updated in August 2014).

Stage

Review completed in March, 2014.
Current stage: Review and meta-analyses completed.

Collaborators

None.

Review methods

Context

Much discussion has revolved around the diverse findings on the complex relationships between one of the leading risk factors globally, alcohol consumption, and the leading cause of death and burden of disease globally, ischaemic heart disease (IHD). While most research to date has focused on average alcohol consumption, there is accumulating evidence that drinking patterns might modify this relationship. Specific risk of episodic heavy drinking in comparison to lifetime abstainers has not been systematically examined before and it is currently unclear whether episodic heavy drinking has a protective, neutral, or detrimental association with IHD.

Primary outcomes

Incidence of IHD events.

Secondary outcomes

Fatal and non-fatal IHD events.

Type of review

Prognostic.

Language

English, Spanish, German.

Country

Canada.

Dissemination plans

Publication in peer-reviewed journal.

Keywords

Alcohol drinking, episodic heavy drinking, ischaemic heart disease, systematic review, meta-analysis

Details of any existing review of the same topic by the same authors

None.

Review status

Completed, but not published.

Review question 1

What systematic evidence exists for the relationship between alcohol consumption and IHD risk?

Literature searches

Using PRISMA guidelines [1], we searched electronic databases from 1980 to second week of August 2014 for meta-analyses on total alcohol consumption and IHD risk (Figure S1).

Search 1

Databases searched: MEDLINE, EMBASE (updated to August 14, 2014)
Search strategy in Medline and Embase (through OVID):

1	alcohol.mp.
2	heart disease.mp.
3	meta-analysis.mp.
4	1 and 2 and 3

Types of studies to be included initially

Meta-analyses.

Strategy for data synthesis

The most comprehensive and recent meta-analysis reporting data by dimensions of alcohol consumption (lifetime drinking status, average consumption, drinking pattern) by sex and IHD endpoint (mortality vs. morbidity) was used in a narrative review.

Review question 2

What is the relative risk for IHD among heavy- and non-heavy alcohol drinkers?

Literature search

Using PRISMA guidelines [1], we searched electronic databases from 1980 to fourth week of March 2014 for original articles, excluding letters, editorials, conference abstracts, reviews, and comments for variations of search terms for the exposure (alcohol consumption), outcome (IHD), and study design from 1980 to fourth week of March 2014 (Figure S2). Additionally, we hand searched references of identified papers and relevant reviews and meta-analyses.

Participants/population

Inclusion criteria: Adults (≥18 years) from population samples, IHD was analyzed as a separate outcome (ICD-9: 410-414, ICD-10: I20-25), case-control or prospective or historical cohort study design, exposure measurement had to cover a reference period of more than 2 weeks for average alcohol consumption at baseline, a drinking group that either specifically excluded or included episodic heavy drinking among current drinkers with an average alcohol consumption <30 g of pure alcohol per day, a measure of risk in comparison to lifetime abstainers and its corresponding measure of variability was reported (or sufficient data to calculate these), and English-, German-, or Spanish-language.

Exclusion criteria: Adolescents (<18 years), population samples from people with IHD-related conditions. We excluded self-reported IHD outcomes, as well as studies reporting estimates on cardiovascular outcomes combined rather than IHD separately and studies with precursors as outcome.

Intervention/exposure

Non-heavy drinking (1-2 drinks on average and usual consumption <30 g/occasion), episodic heavy drinking (1-2 drinks on average and 5+ drinks per occasion or intoxication) are the exposures of interest.

Comparators/controls

Measure of relative risk in comparison to lifetime abstainers in population studies.

Types of studies to be included initially

Observational studies on alcohol consumption and ischaemic heart disease.

Search 2

Databases searched: MEDLINE, EMBASE, Web of Science (Science Citation Index Expanded, Social Sciences Citation Index, Arts & Humanities Citation Index), and ETOH (Alcohol and Alcohol Problems Science Database, National Institute on Alcohol Abuse and Alcoholism, January 1980–December 2003).
Search strategy in Medline (through OVID):

1	human/
2	(comment or editorial or letter or meta-analysis or review).pt.
3	1 not 2
4	(alcohol drinking or alcoholic beverages or heavy drinking occasion* or heavy episodic drinking or binge drinking or alcoholic intoxication or problem drinking or hangover* or irregular drinking or drinking pattern or inebriation).mp.
5	exp drinking behavior/ or exp alcohol drinking/ or exp binge drinking/
6	4 or 5
7	(myocardial ischemia or myocardial infarction or myocardial infarct$ or coronary disease or heart diseases or coronary artery disease or coronary heart disease or angina or cardiac death$ or ischaemic heart disease or ischemic heart disease or cardiac event$ or coronary event$).mp.
8	exp myocardial ischemia/ or exp coronary artery disease/
9	7 or 8
10	exp Case-Control Studies/
11	exp cohort studies/ or exp follow-up studies/ or exp longitudinal studies/ or exp prospective studies/ or exp retrospective studies/
12	exp risk/
13	10 or 11 or 12
14	3 and 6 and 9 and 13
15	limit 14 to yr="1980 - 2014"

Data extraction

From all relevant articles we extracted authors' names, year of publication, country, calendar year(s) of baseline examination, follow-up period, setting, assessment of IHD and alcohol consumption, mean and range of age at baseline, sex, number of observed IHD cases or deaths among participants by drinking group, number of total participants by drinking group, adjustment for potential confounders, and relative risk and its standard error. We used the most adjusted relative risk reported. Information found in related papers from the same cohort was used where possible. The first author performed the literature search and abstracted the data. Full-text articles with uncertain eligibility were discussed by both authors until consensus was reached. Primary authors were not contacted in case there was not enough information presented in the article.

Risk of bias

Most quality scores are tailored for meta-analyses of randomized trials of interventions [2-5] and many criteria do not apply to epidemiological studies like the ones examined here. Also, their use in meta-analyses remains controversial [5, 6]. Thus, quality assessment was incorporated differently by including quality components such as study design and alcohol measurement into the inclusion and exclusion criteria (please see Data abstraction and Table S1 for details). Quality checklists therefore would not have been able to distinguish the quality of selected studies in our analysis.

Strategy for data synthesis

Hazard ratios, odds ratios, and relative risks were treated as equivalent measures of risk. Analyses were stratified by sex where possible. If necessary, relative risks within studies were re-calculated based on the method described by Hamling *et al*. [7] and pooled across studies using inverse-variance weighted DerSimonian-Laird random-effect models to allow for between-study heterogeneity [8]. We quantified between-study heterogeneity using Cochran's Q [9] and the I^2 statistic [10]. I^2 can be interpreted as the proportion of the total variation other than chance that is due

to heterogeneity between studies. We tested for potential publication bias using Egger's test [11]. Sensitivity analyses for the influence of single studies on the pooled relative risks were conducted omitting one study at a time and re-estimating the pooled relative risk. All meta-analytical procedures were conducted on the natural log scale in Stata statistical software, version 12.1 (Stata Corp, College Station, Texas), and $p<0\cdot05$ (two-sided) was considered statistically significant.

Analysis of subgroups or subsets

Subgroup analyses were completed for different classification of alcohol exposure (episodic heavy drinking, non-heavy drinking.

Figure S1. Search results for meta-analyses on alcohol consumption and IHD risk

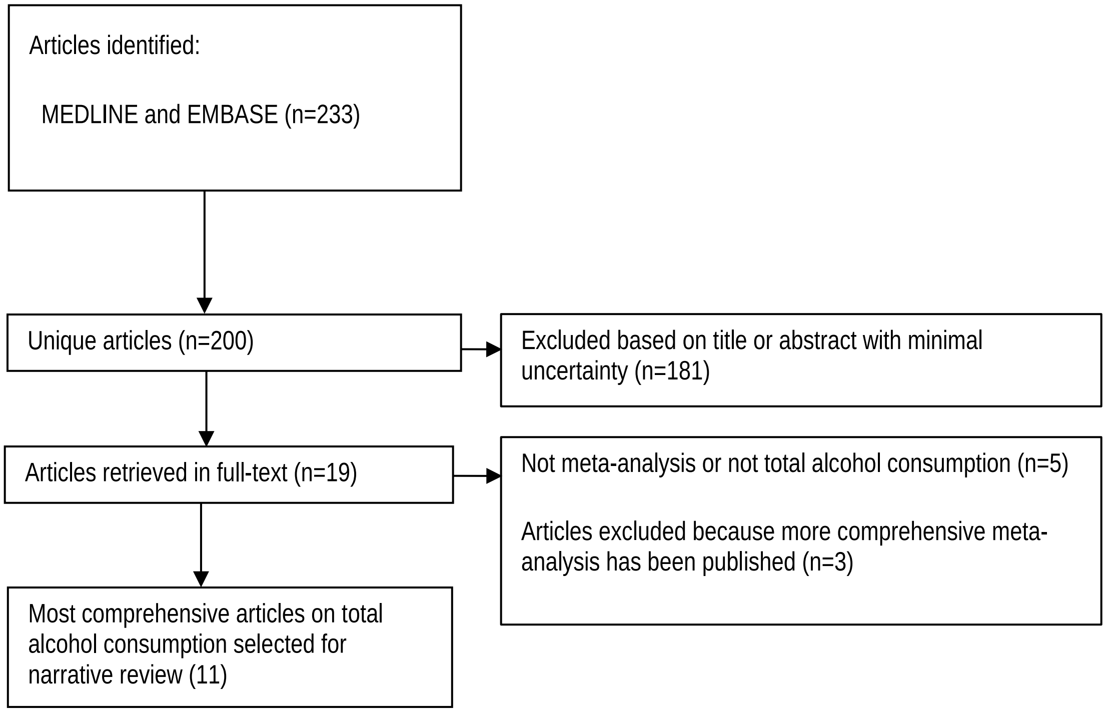

Meta-analyses selected (reference no. from main article):

15. Roerecke M, Rehm J: The cardioprotective association of average alcohol consumption and ischaemic heart disease: a systematic review and meta-analysis. Addiction 2012, 107(7):1246-1260.
16. Corrao G, Rubbiati L, Bagnardi V, Zambon A, Poikolainen K: Alcohol and coronary heart disease: a meta-analysis. Addiction 2000, 95(10):1505-1523.
17. Maclure M: Demonstration of deductive meta-analysis: Ethanol intake and risk of myocardial infarction. Epidemiol Rev 1993, 15:328-351.
18. Ronksley PE, Brien SE, Turner BJ, Mukamal KJ, Ghali WA: Association of alcohol consumption with selected cardiovascular disease outcomes: a systematic review and meta-analysis. BMJ 2011, 342:d671.
19. Brien SE, Ronksley PE, Turner BJ, Mukamal KJ, Ghali WA: Effect of alcohol consumption on biological markers associated with risk of coronary heart disease: Systematic review and meta-analysis of interventional studies. BMJ 2011, 342:d636.
20. Rimm EB, Williams P, Fosher K, Criqui MH, Stampfer MJ: Moderate alcohol intake and lower risk of coronary heart disease: meta-analysis of effects on lipids and haemostatic factors. BMJ 1999, 19(7224):1523-1528.
26. Roerecke M, Rehm J: Irregular heavy drinking occasions and risk of ischemic heart disease: a systematic review and meta-analysis. Am J Epidemiol 2010, 171(6):633-644.
33. Roerecke M, Rehm J: Ischemic heart disease mortality and morbidity in former drinkers: a meta-analysis. Am J Epidemiol 2011, 73(3):245-258.
38. Bagnardi V, Zatonski W, Scotti L, La Vecchia C, Corrao G: Does drinking pattern modify the effect of alcohol on the risk of coronary heart disease? Evidence from a meta-analysis. J Epidemiol Community Health 2008, 62(7):615-619.
39. Roerecke M, Rehm J: Chronic heavy drinking and ischaemic heart disease: a systematic review and meta-analysis. Open Heart 2014, 1(e000135).
42. Roerecke M, Rehm J: Cause-specific mortality risk in alcohol use disorder treatment patients: a systematic review and meta-analysis. Int J Epidemiol 2014, 43(3):906-919.

Figure S2. Search results for population studies on non-heavy and heavy alcohol consumption and IHD risk

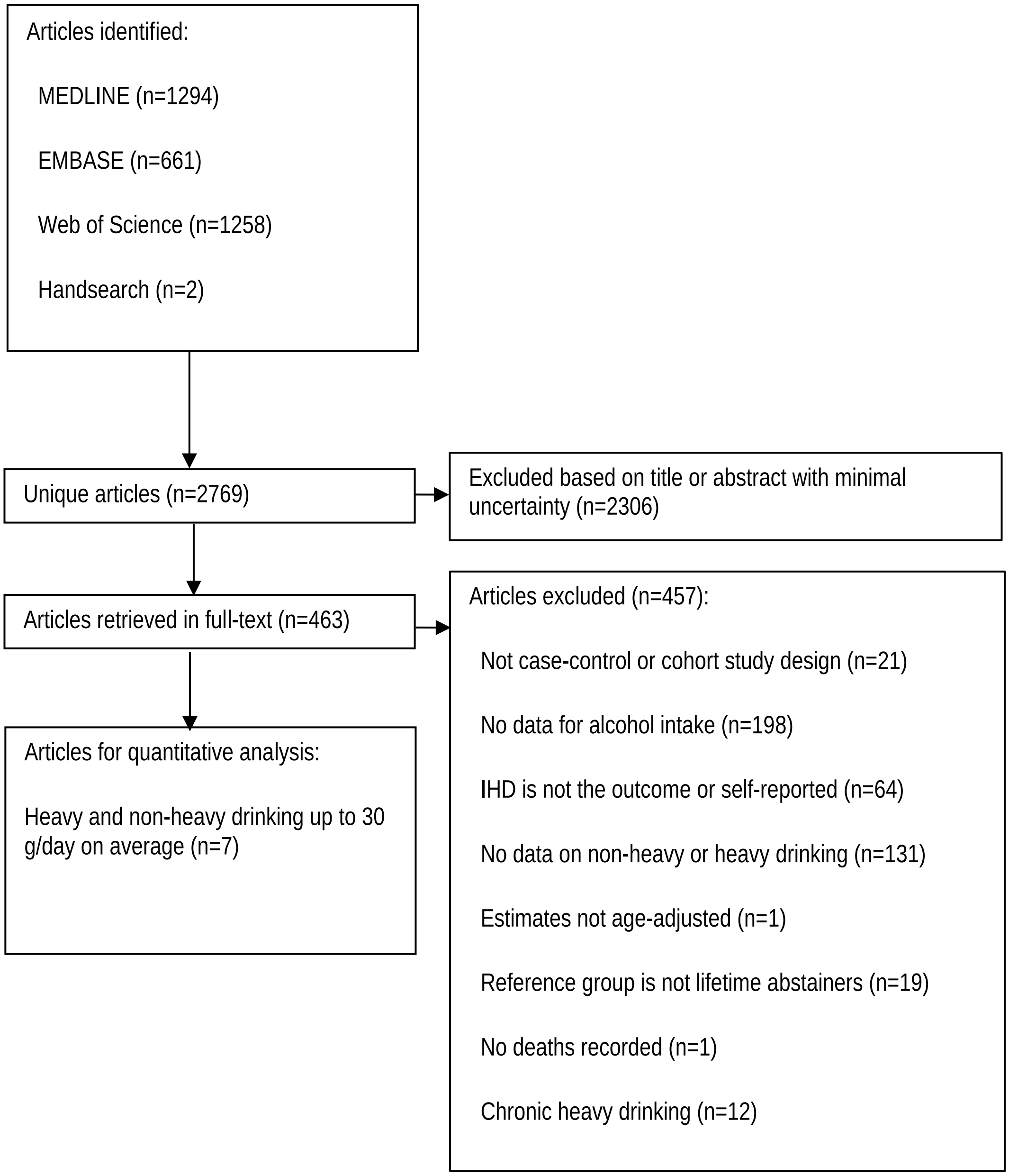

Table S1. Characteristics of 7 studies on IHD risk in drinkers of 1-30 g/day average alcohol consumption in comparison to lifetime abstainers

Study	Cohort	Sex	Sample size*	Reference group	IHD assessment	Non-heavy drinking group	Heavy drinking group	Adjustment
McElduff & Dobson 1997 [12]	MONICA, Newcastle, Australia	W/M	1718 cases, 1099 controls	Non-drinkers (moderate or heavy past drinkers excluded)	Coronary event defined as coronary death (definite fatal MI, possible fatal MI, unclassifiable coronary death), and hospitalized non-fatal MI (WHO criteria)	1 or 2 drinks per drinking day usual consumption	5+ drinks per drinking day usual consumption	Age, smoking, high blood pressure, high cholesterol concentration, angina, stroke, previous MI, diabetes
Wells et al. 2004 [13]	ARCOS (part of the MONICA project), New Zealand	W/M	589 cases, 1149 controls	Never drank >once/month	Coronary event defined as coronary death (definite fatal MI, possible fatal MI, unclassifiable coronary death), and hospitalized non-fatal MI (WHO criteria)	Usual intake <10 -<30 g/occasion	N/A	Age group, history of IHD, smoking, leisure-time physical activity, current anti-hypertensive medication, family history of premature CVD, BMI, diabetes, SES, income, low education
Murray et al. 2006 [14]	Random population sample in Manitoba, Canada	M	59 cases, 1154 at risk	Non-drinkers (0-0.64 g/day), former drinkers excluded	Physician visits, hospital stays, death records from vital statistics file (ICD-9-CM: 410-414)	0.65-18.1 g/day, heavy episodic drinking was excluded	Any ≥ 8 drinks/occasion in previous 12 months	Age, education, marital status, smoking status
Dorn et al. 2007 [15]	Case-control study in Western New York, United States	W	159 cases, 1031 controls	Never drank 12 drinks within 1 year period	First non-fatal MI (WHO criteria), previous MI, coronary bypass graft surgery, percutaneous transluminal coronary angioplasty, symptomatic angina pectoris, previous diagnosis of CVD were ineligible	Not intoxicated previous year	Enough alcohol intake for intoxication previous year	Age, BMI, race, smoking, menopausal status
Mukamal 2010 [16]	NHIS 1987-2002, United States	W	700† cases, 75 533 women at risk	Long-term abstainers	Record linkage with NDI (ICD-9: 410-414, ICD-10: I20-I25)	Light to moderate drinking, no binge drinking (5 or more drinks on one day)	Binge drinking (5 or more drinks on one day)	Age, sex, race, smoking, marital status, education, region, urbanization, BMI, general health status
Roerecke et al. 2011 [17]	National Alcohol Survey 1984 and 1995, United States	W/M	162 cases, 4700 at risk	Less than 12 drinks in lifetime	Record linkage with NDI (ICD-9: 410-414, ICD-10: I20-I25)	Average consumption 2.5-28 g/day (men) 2.5-14 g/day (women); heavy episodic drinking was excluded	Any 5+ drinks/occasion in the previous 12 months	Age, smoking status, race, education, employment status, marital status, income, survey, region, depression symptoms, born outside US, other drug use
Rostron 2012 [18]	NHIS 1997-2004, United States	W/M	2000† cases, 138 000† at risk	Less than 12 drinks in lifetime	Record linkage with NDI (ICD-10: I20-I25)	1 or 2 drinks per drinking day usual consumption	N/A	Race/ethnicity, education, marital status, family income, smoking status, BMI

Abbreviations: ARCOS, Auckland Region Coronary or Stroke Study; BMI, body mass index; CVD, cardiovascular disease; IHD, ischaemic heart disease; M, men; MONICA, Monitoring Trends and Determinants in Cardiovascular Disease; MI, myocardial infarction; N/A, not applicable; NDI, National Death Index; NHIS, National Health Interview Survey; SES, socio-economic status; W, women; WHO, World Health Organization. Age was the time variable in Rostron 2012. Very infrequent (occasional) drinkers were excluded.

* Used in the analysis.

† Estimated.

Figure S3. IHD risk among non-heavy drinkers with total average intake of 1-30 g/day compared to lifetime abstainers

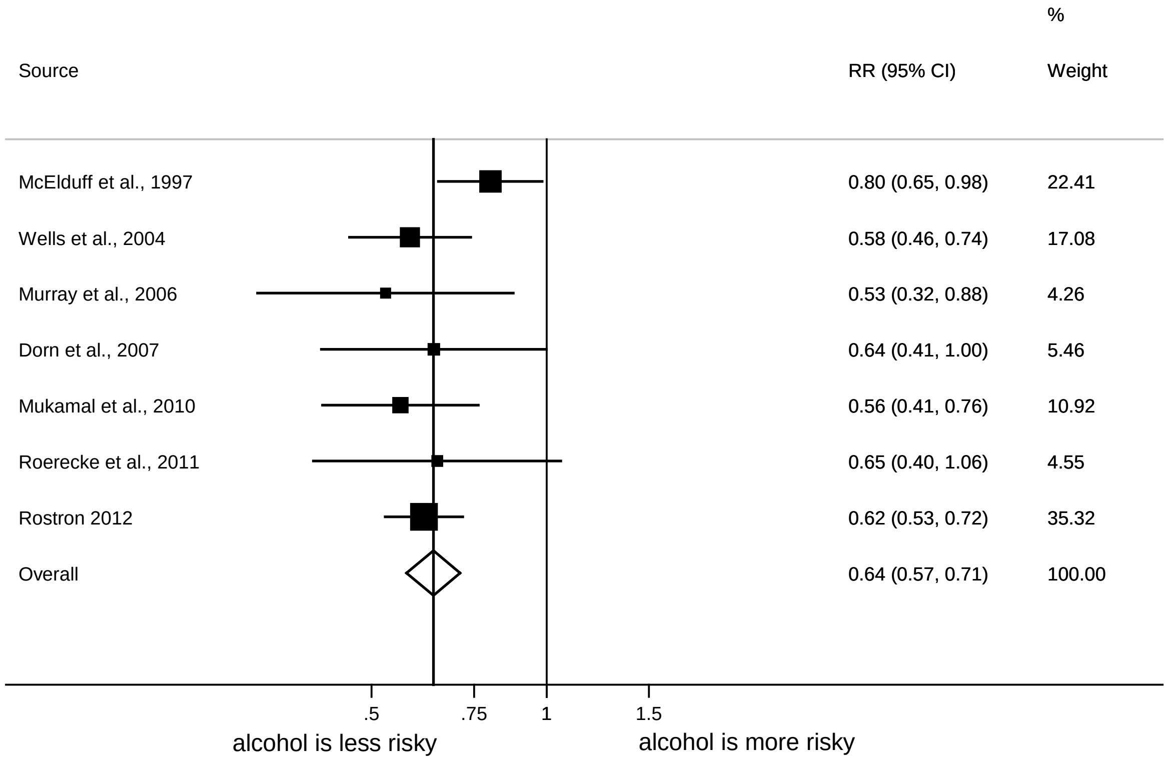

χ^2=6.67, d.f.=6, P=0.35, I^2=10%, publication bias: P=0.62

Figure S4. IHD risk among episodic heavy drinkers with total average intake of 1-30 g/day compared to lifetime abstainers

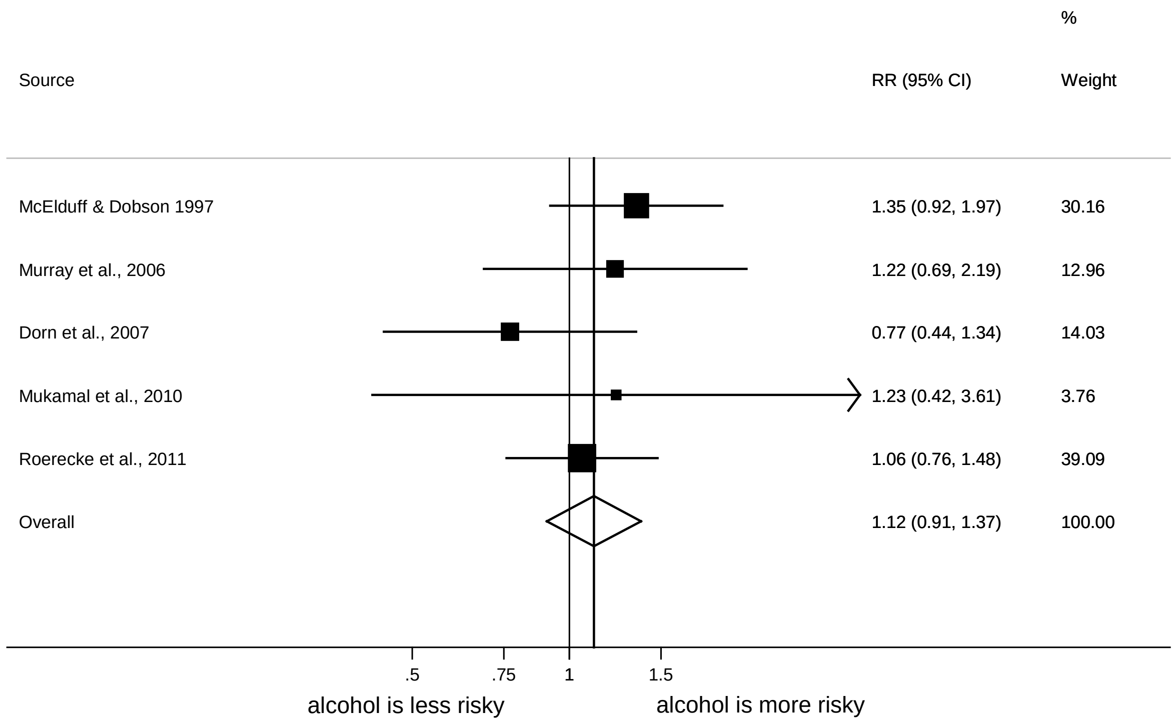

χ^2=2.86, d.f.=4, *P*=0.58, I^2=0%, publication bias: *P*=0.58

Reference List

1. Moher D, Liberati A, Tetzlaff J, Altman DG, The Prisma Group: **Preferred reporting items for systematic reviews and meta-analyses: The PRISMA Statement**. *PLoS Med* 2009, **6**(6):e1000097.
2. Moher D, Pham B, Jones A, Cook DJ, Jadad AR, Moher M, Tugwell P, Klassen TP: **Does quality of reports of randomised trials affect estimates of intervention efficacy reported in meta-analyses?** *Lancet* 1998, **352**(9128):609-613.
3. Chalmers TC, Smith HJ, Blackburn B, Silverman B, Schroeder B, Reitman D, Ambroz A: **A method for assessing the quality of a randomized control trial**. *Control Clin Trials* 1981, **2**(1):31-49.
4. Detsky AS, Naylor CD, O'Rourke K, McGreer AJ, L'Abbé KA: **Incorporating variations in the quality of individual randomized trials into meta-analysis**. *J Clin Epidemiol* 1992, **45**(3):255-265.
5. Greenland S, O'Rourke K: **On the bias produced by quality scores in meta-analysis, and a hierarchical view of proposed solutions**. *Biostatistics* 2001, **2**(4):463-471.
6. Herbison P, Hay-Smith J, Gillespie WJ: **Adjustment of meta-analyses on the basis of quality scores should be abandoned**. *J Clin Epidemiol* 2006, **59**(12):1249-1256.
7. Hamling J, Lee P, Weitkunat R, Ambühl M: **Facilitating meta-analyses by deriving relative effect and precision estimates for alternative comparisons from a set of estimates presented by exposure level or disease category**. *Stat Med* 2008, **27**(7):954-970.
8. DerSimonian R, Laird N: **Meta-analysis in clinical trials**. *Control Clin Trials* 1986, **7**(3):177-188.
9. Cochran WG: **The combination of estimates from different experiments**. *Biometrics* 1954, **10**(1):101-129.
10. Higgins JP, Thompson SG: **Quantifying heterogeneity in a meta-analysis**. *Stat Med* 2002, **21**(11):1539-1558.
11. Egger M, Smith GD, Schneider M, Minder C: **Bias in meta-analysis detected by a simple, graphical test**. *BMJ* 1997, **315**(7109):629-634.
12. McElduff P, Dobson AJ: **How much alcohol and how often? Population based case-control study of alcohol consumption and risk of a major coronary event**. *BMJ* 1997, **314**(7088):1159-1164.
13. Wells S, Broad J, Jackson R: **Alcohol consumption and its contribution to the burden of coronary heart disease in middle-aged and older New Zealanders: A population-based case-control study**. *N Z Med J* 2004, **117**(1190).
14. Murray RP, Ekuma O, Barnes GE: **Drinking pattern as a predictor of cardiovascular harm: A longitudinal study using alternative drinking pattern measures**. *Journal of Substance Use* 2006, **11**(5):359-368.
15. Dorn JM, Hovey K, Williams BA: **Alcohol drinking pattern and non-fatal myocardial infarction in women**. *Addition* 2007, **102**(5):730-739.
16. Mukamal KJ, Chen CM, Rao SR, Breslow RA: **Alcohol consumption and cardiovascular mortality among U.S. adults, 1987 to 2002**. *J Am Coll Cardiol* 2010, **55**(13):1328-1335.
17. Roerecke M, Greenfield T, Kerr W, Bondy S, Cohen J, Rehm J: **Heavy drinking occasions in relation to ischaemic heart disease mortality - An 11-22 year follow-up of the 1984 and 1995 US National Alcohol Surveys**. *Int J Epidemiol* 2011, **40**(5):1401-1410.
18. Rostron B: **Alcohol consumption and mortality risks in the USA**. *Alcohol Alcohol* 2012, **47**(3):334-339.
